# *nab*-Sirolimus for Patients With Malignant Perivascular Epithelioid Cell Tumors

**DOI:** 10.1200/JCO.21.01728

**Published:** 2021-10-12

**Authors:** Andrew J. Wagner, Vinod Ravi, Richard F. Riedel, Kristen Ganjoo, Brian A. Van Tine, Rashmi Chugh, Lee Cranmer, Erlinda M. Gordon, Jason L. Hornick, Heng Du, Berta Grigorian, Anita N. Schmid, Shihe Hou, Katherine Harris, David J. Kwiatkowski, Neil P. Desai, Mark A. Dickson

**Affiliations:** ^1^ Dana-Farber Cancer Institute and Harvard Medical School, Boston MA; ^2^ MD Anderson Cancer Center, Houston, TX; ^3^ Duke Cancer Institute, Durham, NC; ^4^ Stanford University, Stanford, CA; ^5^ Washington University in St Louis, St Louis, MO; ^6^ University of Michigan, Ann Arbor, MI; ^7^ University of Washington/Fred Hutchinson Cancer Research Center, Seattle, WA; ^8^ Sarcoma Oncology Center, Santa Monica, CA; ^9^ Brigham and Women's Hospital, Boston, MA; ^10^ Aadi Bioscience Inc, Pacific Palisades, CA; ^11^ Memorial Sloan Kettering Cancer Center and Weill Cornell Medical College, New York, NY

## Abstract

Malignant perivascular epithelioid cell tumor (PEComa) is a rare aggressive sarcoma, with no approved treatment. To our knowledge, this phase II, single-arm, registration trial is the first prospective clinical trial in this disease, investigating the safety and efficacy of the mammalian target of rapamycin inhibitor *nab*-sirolimus (AMPECT, NCT02494570).

**PATIENTS AND METHODS:**

Patients with malignant PEComa were treated with *nab*-sirolimus 100 mg/m^2^ intravenously once weekly for 2 weeks in 3-week cycles. The primary end point was objective response rate evaluated by independent radiology review. Key secondary end points included duration of response, progression-free survival, and safety. A key exploratory end point was tumor biomarker analysis.

**RESULTS:**

Thirty-four patients were treated (safety evaluable), and 31 were evaluable for efficacy. The overall response rate was 39% (12 of 31; 95% CI, 22 to 58) with one complete and 11 partial responses, 52% (16 of 31) of patients had stable disease, and 10% (3 of 31) had progressive disease. Responses were of rapid onset (67% by week 6) and durable. Median duration of response was not reached after a median follow-up for response of 2.5 years, with 7 of 12 responders with treatment ongoing (range, 5.6-47.2+ months). Twenty-five of 31 patients had tumor mutation profiling: 8 of 9 (89%) patients with a *TSC2* mutation achieved a confirmed response versus 2 of 16 (13%) without *TSC2* mutation (*P* < .001). The median progression-free survival was 10.6 months (95% CI, 5.5 months to not reached), and the median overall survival was 40.8 months (95% CI, 22.2 months to not reached). Most treatment-related adverse events were grade 1 or 2 and were manageable for long-term treatment. No grade ≥ 4 treatment-related events occurred.

**CONCLUSION:**

*nab*-Sirolimus is active in patients with malignant PEComa. The response rate, durability of response, disease control rate, and safety profile support that *nab*-sirolimus represents an important new treatment option for this disease.

## INTRODUCTION

Perivascular epithelioid cell tumors (PEComas) are mesenchymal neoplasms, composed of histologically and immunohistochemically distinctive epithelioid cells.^1,2^ Most PEComas are clinically benign and do not metastasize, but malignant PEComas demonstrate local invasion and/or metastatic spread. Malignant PEComas are classified as an ultrarare soft tissue sarcoma (STS) with an estimated annual incidence of ≤ 1/1,000,000 population,^3^ arise most commonly at visceral sites (especially renal, uterine, and gastrointestinal), and have a female predominance.

**CONTEXT**

**Key Objective**
Malignant perivascular epithelioid cell tumor is a rare aggressive soft tissue sarcoma, with no approved treatment. To our knowledge, the AMPECT study is the first prospective clinical trial in this disease and evaluated the efficacy and safety of the novel mammalian target of rapamycin inhibitor *nab*-sirolimus.
**Knowledge Generated**
The overall response rate to *nab*-sirolimus was 39%, with one complete and 11 partial responses, exceeding the prespecified lower-bound objective response rate of 15% below which the regimen would be considered no more active than standard doxorubicin-based chemotherapy. Responses were of rapid onset and durable. Given the aggressive natural history of the disease not known to spontaneously regress, the responses are most likely due to antitumor activity of *nab*-sirolimus. The median progression-free survival was 10.6 months, and the progression-free survival rate at 6 months was 70%, significantly exceeding the benchmark (14%) that is considered potentially active in advanced soft tissue sarcoma.
**Relevance**
*nab*-Sirolimus may offer an important benefit and a new treatment option in a rare and aggressive sarcoma, perivascular epithelioid cell tumor.

Malignant PEComa has no approved treatment. Although often treated with cytotoxic chemotherapy regimens, these have shown modest benefit.^4^ Some patients with PEComas benefited from treatment with mTORC1 inhibitors (including sirolimus, everolimus, and temsirolimus), as described in case reports and retrospective analyses.^4-9^ PEComas commonly have loss-of-function mutations in or deletions of *TSC1* or *TSC2*.^10^ In addition, PEComas often show evidence of mTORC1 activation with phosphorylation of p70S6K and ribosomal protein S6 by immunohistochemistry (IHC).^11^ Aberrant mTORC1 signaling is a key driver of cell proliferation and tumor formation,^12^ suggesting that mTORC1 inhibition may be a promising therapeutic approach for PEComas.

The orally available mammalian target of rapamycin (mTOR) inhibitors sirolimus and everolimus have variable absorption, often require therapeutic drug monitoring, and have incomplete target suppression.^13-15^ *nab*-Sirolimus (nanoparticle albumin–bound sirolimus; ABI-009, formerly known as *nab*-rapamycin) is a novel intravenous (IV) mTOR inhibitor with significantly higher tumor growth inhibition, higher intratumoral drug accumulation, and greater mTOR target [phospho-S6 (pS6)] suppression compared with oral inhibitors, as demonstrated in preclinical models.^16^

To our knowledge, this trial (AMPECT) is the first prospective clinical trial in advanced malignant PEComa. Herein, we describe the safety and efficacy of *nab*-sirolimus in patients with this disease.

## PATIENTS AND METHODS

### Patients

Eligible adults (age ≥ 18 years) had an Eastern Cooperative Oncology Group performance status score ≤ 1, had not previously received an mTOR inhibitor, and had a histologically confirmed diagnosis of either metastatic or locally advanced (ineligible for surgery) malignant PEComa and measurable disease according to the RECIST, v1.1. Histology was assessed locally in each institution at enrollment and subsequently confirmed by central review at the Dana-Farber/Harvard Cancer Center (J.L.H.). Pathologic confirmation of PEComa was based on characteristic histologic features and evidence of melanocytic (HMB-45 and/or melan A) and smooth muscle (smooth muscle actin and/or desmin) differentiation by IHC; PEComas with either marked nuclear atypia or pleomorphism combined with mitotic activity were considered malignant.^1^

Eligible patients had adequate hematologic, hepatic, and renal function, including an absolute neutrophil count of ≥ 1.5 × 10^9^/L, platelets of ≥ 100 × 10^9^/L, a hemoglobin level of ≥ 9 g/dL, a bilirubin level of ≤ 1.5 × upper limit of normal, and a serum creatinine level of ≤ 1.5 × upper limit of normal.

### Trial Design and Treatment

In this multicenter, open-label, phase II registration study, patients received *nab*-sirolimus 100 mg/m^2^ IV over 30 minutes once on days 1 and 8 of a 21-day cycle. A maximum of two dose reductions to 75 and 56 mg/m^2^ were permitted for toxicity. Treatment continued until disease progression, unacceptable toxicities, or patient preference.

### End Points and Statistical Analysis

The primary efficacy end point was overall objective response rate (ORR) by 6 months, evaluated by independent radiology review. The primary analysis was preplanned to occur when the last enrolled patient had been treated for 6 months. The sample size estimation assumed an observed 30% ORR and a sample size of 30 patients, which would exclude values < 15% for the lower bound of the 95% CI. Secondary end points included duration of response (DOR), progression-free survival (PFS), PFS at 6 months (PFS6), overall survival (OS), and safety. A key exploratory end point evaluated the association of tumor mutational and biomarker analyses with clinical response.

The DOR, PFS, and OS reported here are based on an additional 1.5-year follow-up after the primary analysis date.

### Assessments

All patients receiving at least one dose of *nab*-sirolimus were evaluable for toxicity. All adverse events (AEs) were collected from the time the patient signed informed consent until 28 days after the last dose of *nab*-sirolimus. AEs were graded by National Cancer Institute Common Terminology Criteria for Adverse Events v4.03 and were coded using the Medical Dictionary for Regulatory Activities.

Patients were evaluated by contrast-enhanced computed tomography or magnetic resonance imaging if computed tomography was contraindicated every 6 weeks for the first year and then every 12 weeks until disease progression. Patients evaluable for efficacy had ≥ 1 dose of *nab*-sirolimus and centrally confirmed PEComa.

Tumor response was evaluated by RECIST v1.1 by investigators and independent review by two radiologists and an adjudicator, who were unaware of the investigators' assessment.

Patients were followed for survival every 12 weeks until death, loss to follow-up, or study closure.

### Biomarker Study Methodology

Targeted exome next-generation sequencing using the OncoPanel test (Center for Advanced Molecular Diagnostics, Brigham, and Women's Hospital, Boston, MA)^17^ was performed to assess mutations, copy number changes, and translocation events in approximately 500 genes. In addition, pS6, phospho-4EBP1 (p4EBP1), SPARC, %Ki67, and percent of cleaved caspase 3 were assessed by IHC, and *TFE3* translocation by fluorescence in situ hybridization.

Mutational and biomarker analyses were blinded with respect to clinical outcome.

A multivariable analysis was conducted using Pearson correlation to correlate clinical response with altered genes and the following biomarkers: TFE3, phospho-AKT, pS6, p4EBP, SPARC, Ki67, and cleaved caspase 3.

### Trial Oversight

The study was approved by the institutional review board of each participating site and was conducted in accordance with the International Conference on Harmonization requirements for Good Clinical Practice and with the ethical principles outlined in the Declaration of Helsinki. All patients provided written informed consent before the initiation of the study.

Independent Data Monitoring Committee meetings were convened when 14 and 26 patients had completed ≥ 1 cycle of therapy and reported no concerns regarding the safety of *nab*-sirolimus requiring study modification or intervention.

## RESULTS

### Patient Characteristics and Demographics

A total of 35 patients were enrolled between April 2016 and November 2018 at nine community and academic centers across the United States; 34 of 35 patients were treated with at least one dose of *nab*-sirolimus, and 31 were evaluable for efficacy (two did not have PEComa on central pathology review; one did not have sufficient tissue for review). The median age was 60 years (range, 27-78), 82% (25 of 31) were female, and 85% (26 of 31) had metastatic disease (Table 1). The most common primary sites of disease were the uterus (24%), pelvis and retroperitoneum (18% each), and lung and kidney (12% each; Table 1). Thirteen percent (4 of 31) of efficacy-evaluable patients received prior chemotherapy for advanced disease, including gemcitabine-docetaxel, doxorubicin-ifosfamide, and doxorubicin-olaratumab.

**TABLE 1.**
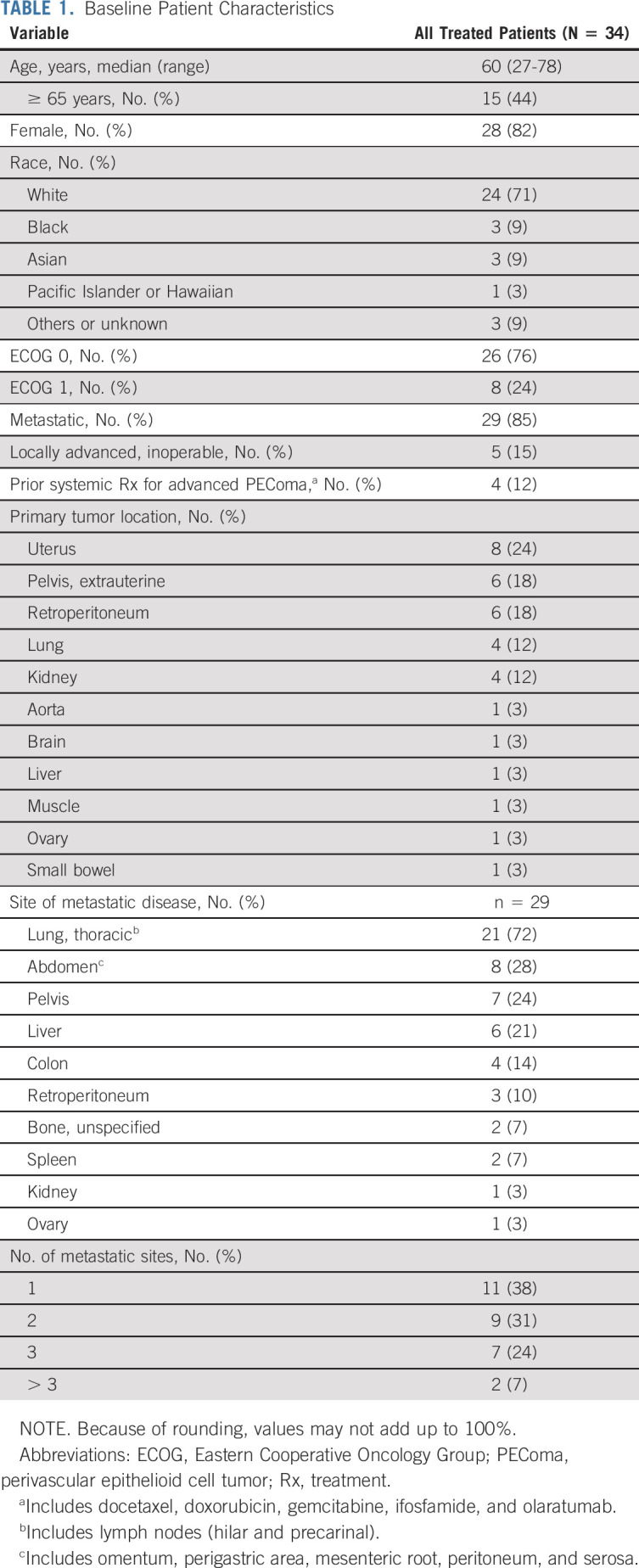
Baseline Patient Characteristics

### Efficacy

#### Response evaluation at the primary analysis.

The primary analysis was preplanned to occur when the last patient enrolled had been treated for 6 months (May 22, 2019). The confirmed ORR as assessed by independent radiologists was 39% (12 of 31; 95% CI, 22 to 58), all partial responses (PRs). One additional patient had an unconfirmed PR without subsequent confirmatory scans and was assessed as stable disease (SD) ≥ 12 weeks. SD occurred in 52% of patients (16 of 31, with 10 of 31 SD ≥ 12 weeks), and 10% of patients had progressive disease (3 of 31; Table 2). The disease control rate (defined as complete response [CR] + PR + SD ≥ 12 weeks) was 71% (22 of 31).

**TABLE 2.**
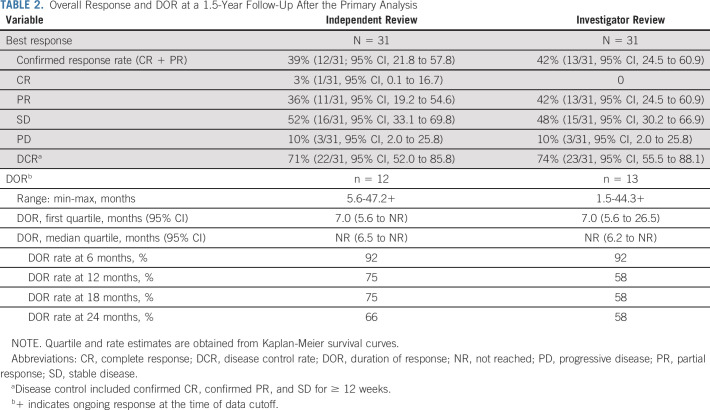
Overall Response and DOR at a 1.5-Year Follow-Up After the Primary Analysis

Responses were of rapid onset and durable. Sixty-seven percent (8 of 12) of PRs were seen at the first scan after baseline at week 6 (median 1.4 months; 95% CI, 1.3 to 2.8). The median DOR was not reached at the time of the primary analysis, with 9 of 12 responders still on treatment.

#### 1.5-year follow-up after the primary analysis for DOR, PFS, and OS.

##### Reponses and DOR

At a 1.5-year follow-up after the primary analysis date (November 23, 2020; ie, 2 year after the last patient initiated treatment), 7 of 12 responders were still receiving treatment and the median DOR had not been reached after a median follow-up for response of 2.5 years (DOR range, 5.6 to 47.2+ months, Table 2).

**FIG 1.**
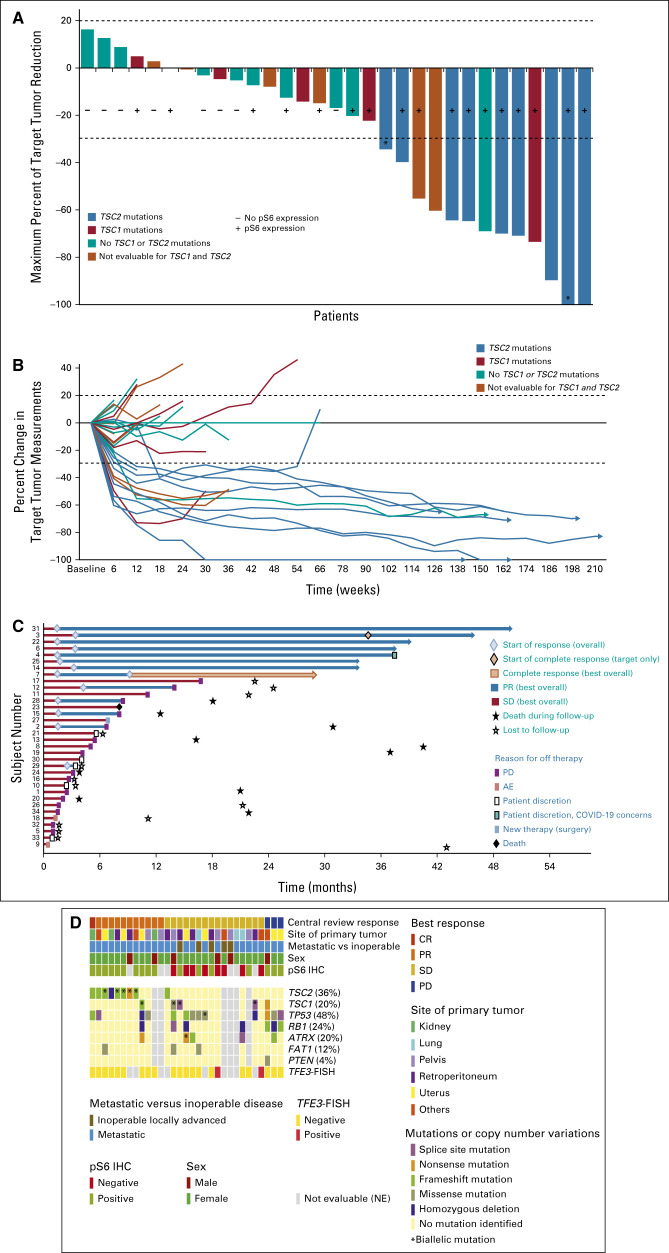
Response to *nab*-sirolimus in patients with PEComa. (A) Waterfall plot of maximum reduction in sum of longest diameters of target tumors, evaluated at the 1.5-year follow-up after the primary analysis. *A patient with unconfirmed PR is considered having SD as best response per RECIST v1.1 and a patient with a complete response of target tumor reduction has a PR as best response because of unresolved nontarget lesions. (B) Spider plot showing change in the sum of target tumor measurements over time. Arrowheads indicate patients who were still on treatment at the time of the 1.5-year follow-up. (C) Swimmer plot showing the treatment duration and response to treatment of individual patients, including reasons for off therapy and survival. (D) Co-Mut plot showing correlation between mutational status and other biomarkers, and response. Each column represents a different patient. Response, clinical features, and pS6 staining by IHC are shown at the top. Then, relevant genes, mutation frequency, and type are shown. Six patients had tumors NE for mutational status because of inadequate tumor sample; PRs occurred in two patients (33%) of this group. CR, complete remission; FISH, fluorescence in situ hybridization; IHC, immunohistochemistry; NE, not evaluable; PEComa, perivascular epithelioid cell tumor; PR, partial response; SD, stable disease.

Figures 1A-1C show the target tumor responses (waterfall plot) and changes over time (spider plot). Notably, one patient with a primary renal PEComa metastatic to the lungs and lymph nodes had a PR for 10 months that converted to a CR (Table 2), with response ongoing at 21.6+ months. One additional patient had a CR in target lesion measurement; however, this patient still had an observed nontarget lesion and an overall assessment of PR.

Responses were independent of the primary site and were observed in tumors originating in the uterus (three), kidney (three), retroperitoneum (two), pelvis (two), liver (one), and small bowel (one; Fig 1D). Notably, 43% (3 of 7) of patients with uterine PEComa had a PR. Responses were also observed in 3 of 4 patients who had previously received chemotherapy, with ongoing DOR ranging from 31.5+ to 47.2+ months.

##### PFS

**FIG 2.**
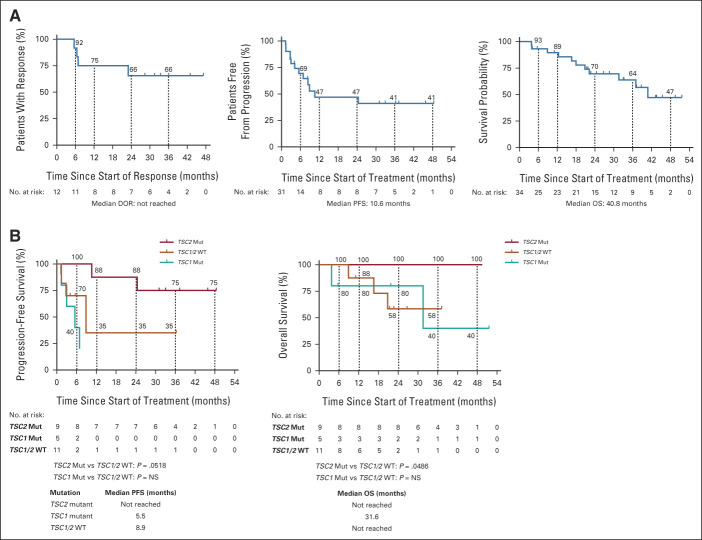
(A) Kaplan-Meier curves for DOR, PFS, and OS for all patients and (B) PFS and OS by mutational status. DOR, duration of response; OS, overall survival; PFS, progression-free survival; WT, wildtype.

The median PFS was 10.6 months (95% CI, 5.5 months to not reached; Fig 2A). The PFS rates at 3, 6, 12, and 24 months were 79%, 69%, 47%, and 47%, respectively.

Two of five (40%) patients with locally advanced disease deemed not eligible for surgery at study entry by the investigators had resection of residual PEComa after treatment with *nab*-sirolimus. Before surgery, one patient had a 7.9% reduction in target lesions after one cycle and the other patient had a 22.3% reduction in target lesions after 10 cycles. Following surgery, both patients remained without disease recurrence at 3 and 3.5 years.

##### OS

The median OS was 40.8 months (95% CI, 22.2 months to not reached), and 23 of 34 treated patients were still alive with OS rates at 6, 12, and 24 months of 93%, 89%, and 70%, respectively (Fig 2A). The median follow-up was 22 months (min, max: 1, 52).

### AEs and Dose Reductions

Most treatment-related adverse events (TRAEs) were grade 1 or 2. No grade 4 or 5 TRAEs occurred. The most common nonhematologic TRAEs (Table 3) were mucositis (79%, 27 of 34 patients), fatigue (59%, 20 of 34), and rash (56%, 19 of 34). The most common hematologic TRAEs were anemia (47%, 16 of 34) and thrombocytopenia (32%, 11 of 34). Noninfectious pneumonitis occurred in 18% (6 of 34) of patients and was grade 1 or 2. Two patients discontinued therapy because of a TRAE (grade 2 anemia and grade 1 cystitis). One patient discontinued therapy because of a fatal AE (upper GI hemorrhage unrelated to treatment).

**TABLE 3.**
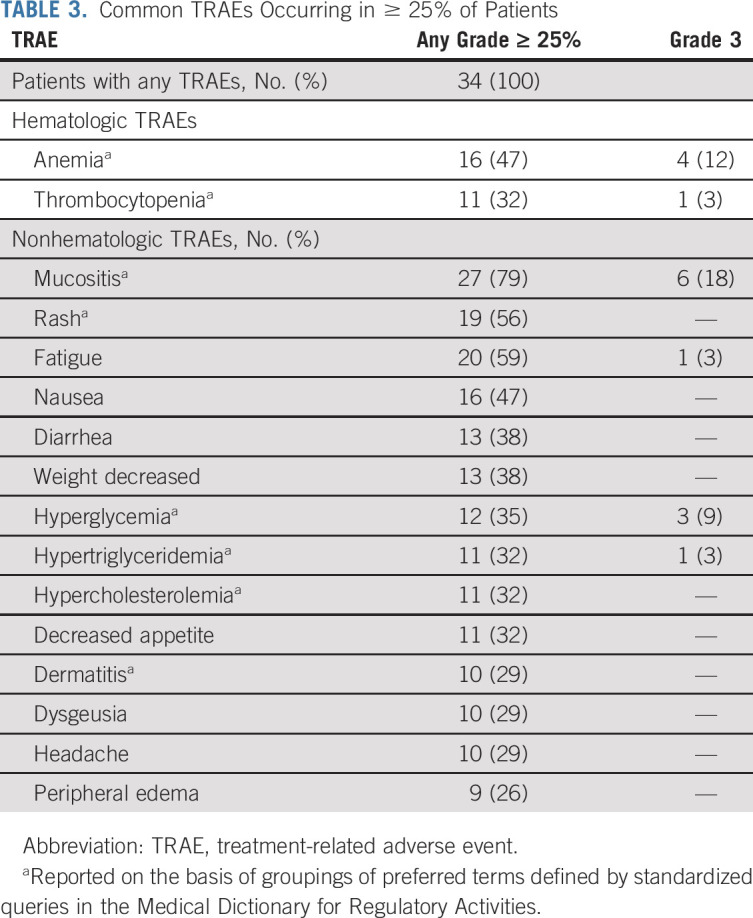
Common TRAEs Occurring in ≥ 25% of Patients

Twenty-four percent (8 of 34) of patients had treatment-related serious adverse events (TRSAEs), with 12 events in total. Most TRSAEs were in metabolism and nutrition disorders (33%, 4 of 12 events: four events of grade 3 dehydration occurring in two patients) and GI disorders (25%, 3 of 12: grade 2 abdominal pain, grade 2 diarrhea, and grade 3 enteritis, occurring in one patient each). Other TRSAEs were acute kidney injury, acute coronary syndrome, edema, and pancytopenia, all grade 3, occurring in one patient each.

Dose reductions occurred in 34% (13 of 34) of patients; 11 of 13 patients had one dose reduction, and two patients had two dose reductions. The most common reasons for dose reductions were mucositis and pneumonitis. In all cases, patients whose doses were reduced maintained their best response at the lower dose.

### Biomarkers

**FIG 3.**
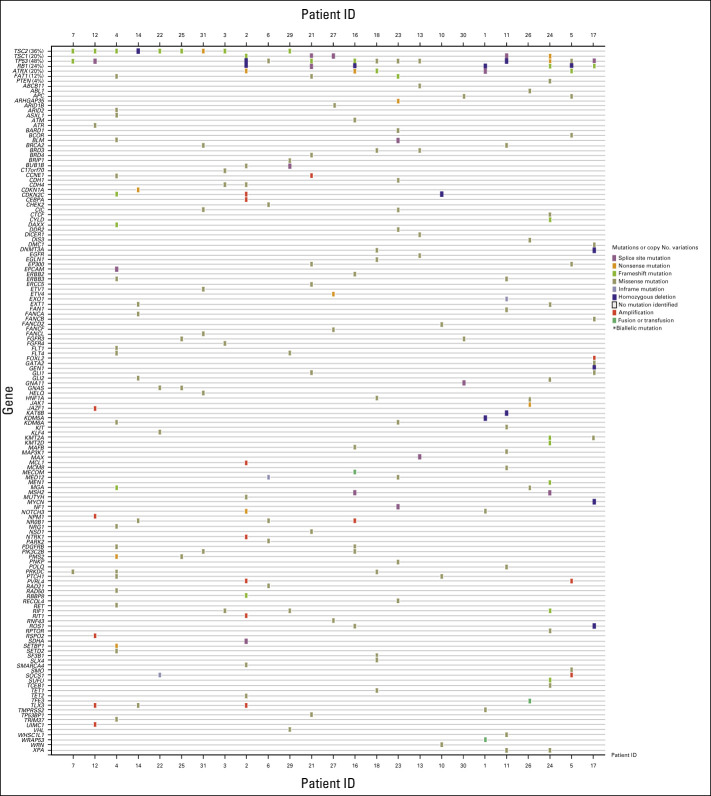
Next-generation sequencing of 25 patient samples.

Twenty-five patients had tissue sufficient for mutational analysis, 25 had tissue analyzed by IHC, and 22 were evaluable for fluorescence in situ hybridization (Figs 1D and 3). Mutation results for seven genes selected on the basis of frequency of alteration or previous studies are shown: *TSC2*, *TSC1*, *TP53*, *RB1*, *ATRX*, *FAT1*, and *PTEN*.^8^

*TSC2* mutations or deletions were seen in 36% (9 of 25) of patients, *TSC1* in 20% (5 of 25), *TP53* in 48% (12 of 25), and *RB1* in 24% (6 of 25; Fig 1D).

On the basis of a multivariate analysis, only *TSC2*-inactivating mutations (*P* < .001, *r*^2^ = 0.560) and pS6 expression (*P* = .004, *r*^2^ = 0.314) were associated with response to *nab*-sirolimus. Eighty-nine percent (8 of 9) of patients with a *TSC2* mutation achieved a confirmed response versus 13% (2 of 16) without a *TSC2* mutation (*P* < .001, Fisher's exact). Of note, 1 of 9 patients with a *TSC2* mutation had an unconfirmed PR; per RECIST v1.1, this patient's overall response was considered SD. Responses occurred in 59% (10 of 17) of patients with pS6+ tumors versus 0 of eight patients with pS6− tumors; the absence of pS6 staining was a negative predictor of response to *nab*-sirolimus (*P* = .008, Fisher's exact). Ninety-one percent (10 of 11) of PEComas with *TSC1* or *TSC2* mutations were pS6+, whereas only 44% (5 of 11) without *TSC1* or *TSC2* mutations were pS6+ (Fisher's exact *P* = .06).

Additional confirmed PRs were seen in 20% (1 of 5) of patients with a *TSC1* mutation, and in 9% (1 of 11) without a mutation in *TSC1* or *TSC2*. Confirmed PRs were also observed in two of the six patients with tumors with unknown mutational status because of insufficient archival material for analysis.

*TSC1* and *TSC2* mutations were mutually exclusive. *TSC2* mutations were not associated with specific anatomic sites: the primary sites of tumors for the nine patients with *TSC2* mutations were retroperitoneum (three), kidney (two), uterus (two), liver (one), and small bowel (one). One of the seven patients with *RB1* mutation responded to *nab*-sirolimus, whereas 9 of 18 patients without *RB1* mutation responded (Fisher's exact *P* = .18).

At a 1.5-year follow-up after the primary analysis date, the median DOR had not been reached for *TSC2* mutations after a median follow-up for response of 33.7 months (6 of 8 ongoing, range: 6.5 to 47.2+ months). *TSC2* mutational status was significantly associated with longer PFS and OS (medians not reached, Fig 2B). One patient with a *TSC1* mutation and one patient with no *TSC1* or *TSC2* mutations had the DOR of 5.6 months and 33.4+ months, respectively.

## DISCUSSION

The AMPECT study met its primary end point with an independently assessed ORR of 39% (95% CI, 22 to 58), exceeding the prespecified lower-bound ORR of 15% below which the regimen would be considered no more active than standard doxorubicin-based chemotherapy. The response rate, durability of response, disease control rate, and toxicity profile support that *nab*-sirolimus may represent an important new treatment option for patients with advanced malignant PEComa.

It is inherently difficult to perform randomized studies for ultrarare indications; thus, this study was a single-arm phase II study to estimate the response rate. There are no prior prospective trials in patients with malignant PEComa, but STS response rates are low and typically < 20%.^18-20^ For example, in a recent study that included doxorubicin as control for an unselected patient population of STS, the response rate was 11.9%.^21^ Although the small sample size in the present study provided a wide confidence interval for the 39% ORR, given the aggressive natural history of the disease not known to spontaneously regress, the responses are most likely due to antitumor activity of *nab*-sirolimus.

Therapeutics yielding PFS rates of ≥ 40% at 3 months and ≥ 14% at 6 months are considered to be potentially active in advanced STS.^22^ A retrospective analysis of cytotoxic chemotherapy in malignant PEComa described a median PFS of 3.2-5.4 months.^4^ In the current study, *nab*-sirolimus significantly exceeded these benchmarks with PFS rates at 3 and 6 months of 79% and 70%, respectively. Although patients with tumors with *TSC2* mutations had a longer PFS and OS compared with those without *TSC2* mutations, we cannot differentiate between this being an effect of *nab-*sirolimus or potentially reflecting variations in clinical behavior of these genotypes. Overall, these outcomes for a targeted therapy are promising and demonstrate the importance of studying the molecular genetics of each type of sarcoma and other cancer types.

A subset of malignant PEComas are associated with mutations (inactivation or deletions) of *TSC1* or *TSC2*, negative regulators of the mTOR signaling pathway. Retrospective analyses of patients with advanced PEComa treated with mTOR inhibitors sirolimus, temsirolimus, or everolimus showed evidence of antitumor activity,^4-8^ suggesting that mTOR inhibitors may improve outcomes compared with chemotherapy and tyrosine kinase inhibitors. *nab*-Sirolimus is a novel albumin-bound mTOR inhibitor characterized by high tumor uptake and mTOR target suppression and may enhance tumor penetration and accumulation via albumin receptor–mediated (gp60) endothelial transcytosis.^23,24^ Albumin has long plasma half-life and broad binding affinity and accumulates in tumors, areas of inflammation, and tissue remodeling, making it an ideal candidate for drug delivery.^23,25^ *nab*-Paclitaxel was the first therapeutic agent using this technology and was shown to have greater drug delivery, safety, and efficacy compared with conventional solvent-based paclitaxel in different various solid tumors.^26,27^ Similarly, *nab*-sirolimus has a distinct pharmacologic profile and pharmacokinetics compared with sirolimus and other mTOR inhibitors,^14,28^ although a direct clinical comparison has not yet been performed.

The female prevalence in this study is consistent with known epidemiology of this disease. The relatively high ORR in patients with primary uterine PEComas is consistent with that of the overall study population and contrasts with retrospective reports of lower sensitivity of uterine PEComas to mTOR inhibitors.^4^

No new safety signals were observed despite relatively high doses of *nab*-sirolimus versus those reported with other mTOR inhibitors.^13,14,29^ The high degree of AE resolution and the ability of patients to continue on therapy for > 2 years suggest that *nab*-sirolimus is manageable for long-term treatment. On the basis of the pharmacokinetic and safety profile of *nab*-sirolimus in a phase I study,^28^ as well as the safety and efficacy in the present study, therapeutic drug monitoring is not required with IV *nab*-sirolimus.

To our knowledge, AMPECT is the first trial to prospectively evaluate treatment outcome and exploratory correlation with mutational status and biomarkers in malignant PEComa. *TSC1*- or *TSC2*-inactivating mutations were seen in 56% (14 of 25) of patients. *TSC2* mutations were a strong positive predictive factor for response to *nab*-sirolimus in these patients, which is consistent with the role of the TSC protein complex in mTORC1 regulation and warrants further studies for the role of *nab*-sirolimus in other tumors with *TSC2*-inactivating mutations. Extensive previous studies have shown that inactivation or loss of either *TSC1* or *TSC2* has similar effects on the activation of mTORC1.^30^ Thus, it remains unclear as to why *TSC2* mutations and not *TSC1* mutations were associated with response in this study. This analysis is limited by the small number of patients (n = 5) with *TSC1*-mutant tumors. Of the four patients with *TSC1*-mutant tumors that did not develop RECIST responses, three had SD, two for at least 12 weeks, suggesting that *nab*-sirolimus might have provided some, but incomplete, antitumor activity in this setting. Other possible explanations could be that the nature of the *TSC1* mutation led to incomplete inactivation of the TSC1/TSC2 complex or other coincident mutations in these tumors such as mutations in *TP53* may contribute to different tumor behavior and outcome (Fig 1D). No significant differences were identified in pharmacokinetic parameters of Cmax or area under the curve in these patients that could account for differences in outcome.

The absence of pS6 staining, which reflects lack of mTORC1 activation, was a strong negative predictor of response to *nab*-sirolimus. *RB1* mutation was also uncommon in responders. Since the total number of patients with mutation and biomarker analysis was relatively small (n = 25), further study of these biomarkers is warranted.

Although other mTOR inhibitors have been used off-label for treatment of advanced malignant PEComa, to our knowledge, the AMPECT study is the first prospective study in this disease and provides evidence that *nab*-sirolimus may offer an important benefit in a rare and aggressive sarcoma for which there are no approved therapies.
